# Nathaniel Gist Gee’s contribution to biology in modern China

**DOI:** 10.1007/s13238-016-0318-x

**Published:** 2016-09-22

**Authors:** Lei Fu

**Affiliations:** Zhejiang Normal University, Jinhua, 321004 China

Nathaniel Gist Gee (祁天锡, 1876–1937) was an American biologist who lived in China for several decades. He greatly contributed to the development of biology in modern China, including the establishment of the first Biology Department in China and introducing the biology graduate education to China.

**Figure 1:**
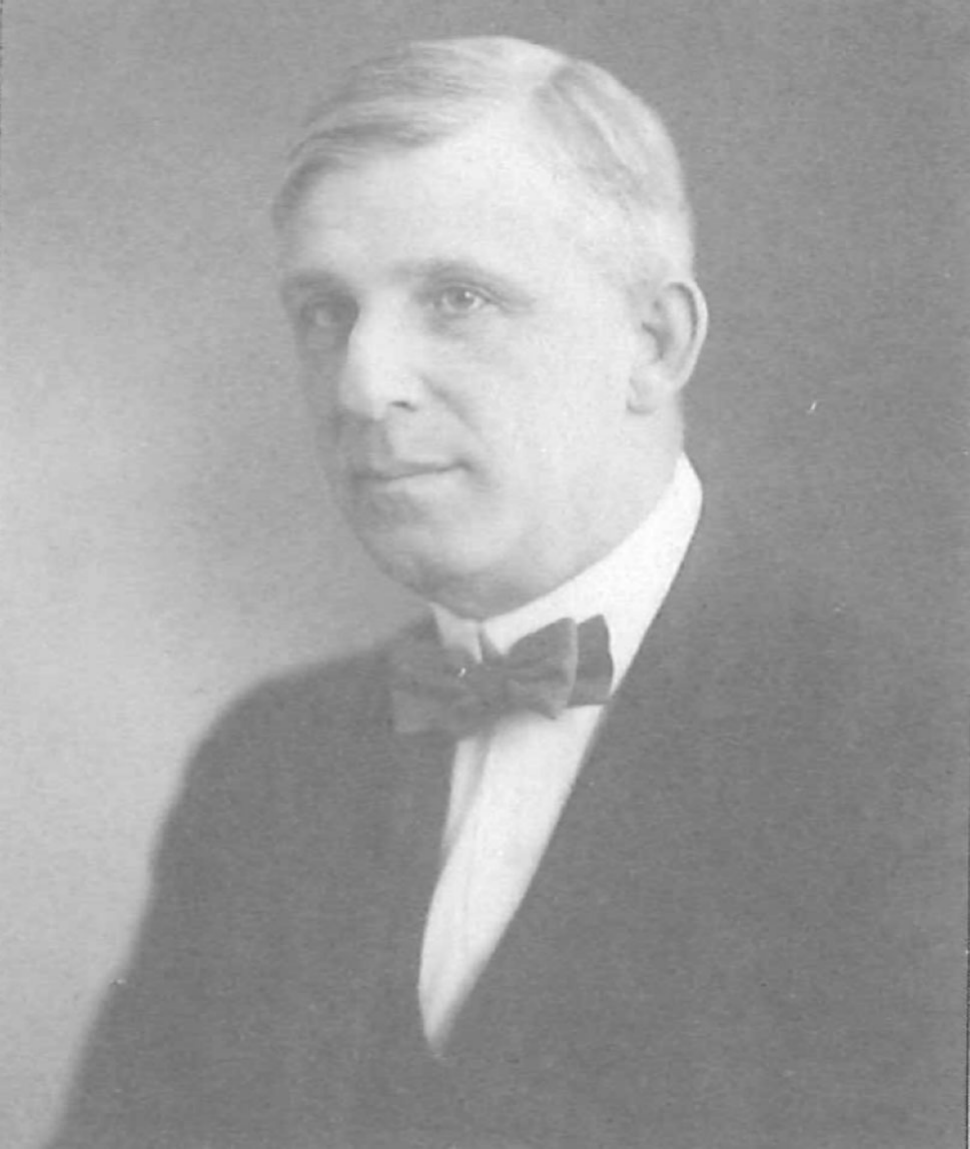
N. Gist Gee in 1933

N. Gist Gee was born in Union, South Carolina, USA on April 20, 1876. He attended Wofford College in 1892 where he received his Master’s Degree. In 1901, after working at Columbia College and other schools, he came to China and was appointed Professor of Natural Sciences in Soochow University. He devoted most of his time to the biology education of China, spending only a few months leave in his native country taking care of his sick wife. He returned to China after his wife’s death in 1921. First serving as representative of the Spencer Lens Company, he was then hired by the China Medical Board of the Rockefeller Foundation. He left China in 1932, and worked as Biology Professor in London College until his death of illness (James, 1938; Wang, 1940).

N. Gist Gee was the first science teacher from abroad who reached the level of university instructor in China. As a biologist, N. Gist Gee promoted the development of biology in modern China, including biology education, investigation of plants and animals, and the institutionalization of biology research.

N. Gist Gee served as a professor in natural sciences at Soochow University from 1901, where he taught biology, physics, chemistry and other subjects for years. Then he became the first Director of Biology Department of this university in 1912, which was the first independent biology department in China. He put a special emphasis on scientific research and founded the most advanced biology laboratory in China at that time. He took students on field trips and led them to pay attention to biological agriculture. He wrote *A Textbook of Botany* for students, and introduced much knowledge about native plants. Shih Jiyan (施季言) and Wu Chenfu Francis (胡经甫) were the first Chinese biology masters cultured in his department and graduated in 1919. After graduation, Shih Jiyan remained working as school superintendent in Soochow University. He was later appointed President of Soochow University, which was reestablished in 1952, in Taiwan. Wu Chenfu Francis received his Ph.D. Degree in 1922 from Cornell University and then returned to China. Wu Chenfu Francis was the founder of modern entomotaxonomy in China (Li, 2012). Many of N. Gist Gee’s students became leaders of biology departments at other universities of China, such as Wu Chenfu Francis at Yenching University, Nelson Chen (陈纳逊) at the University of Nanking, Wang Chu-chia (王志稼) at Shanghai University, Chen Tze Ying (陈子英) at Xiamen University, Chu Yuan-Ting (朱元鼎) at St. John’s University, and so on. These renowned Chinese scientists greatly promoted the development of biology in modern China. The department maintained its excellent education on biology after N. Gist Gee’s leaving the university, and several distinguished biologists and educators were cultivated there, including Harry Zanyi Gaw (高尚荫), Chien Kang Liu (刘建康), and Chia-Chen Tan (谈家桢), et al.

N. Gist Gee also stressed the importance of the popularity of biology and elementary education. He was a biology teacher in the middle school attached to Soochow University for a few years and wrote books for public and middle school students. In 1904, he published *Life Histories of A Few Common Insects*, in which he quoted some Chinese idioms such as *Mantis stalks the cicada oriole in the post* (Gee, 1904). He prepared a biology textbook in English for middle school students named *Introduction to Biology,* published by Commercial Press in 1913. He presented many photos and pictures in this book, some of which were taken at Soochow University and Saint John’s University in Shanghai. In order to help Chinese students, he invited K. K. Woo (吴继杲) to add Chinese technical terms to this textbook (Gee, 1913). He compiled *Science Reader*, *The Useful Knowledge Reader* and other books to introduce the Chinese public the knowledge about native Chinese plants and animals. Furthermore, Gist Gee wrote articles about nature and science for students in magazines published by Commercial Press. All of these publications were very popular and adopted by several schools.

**Figure 2:**
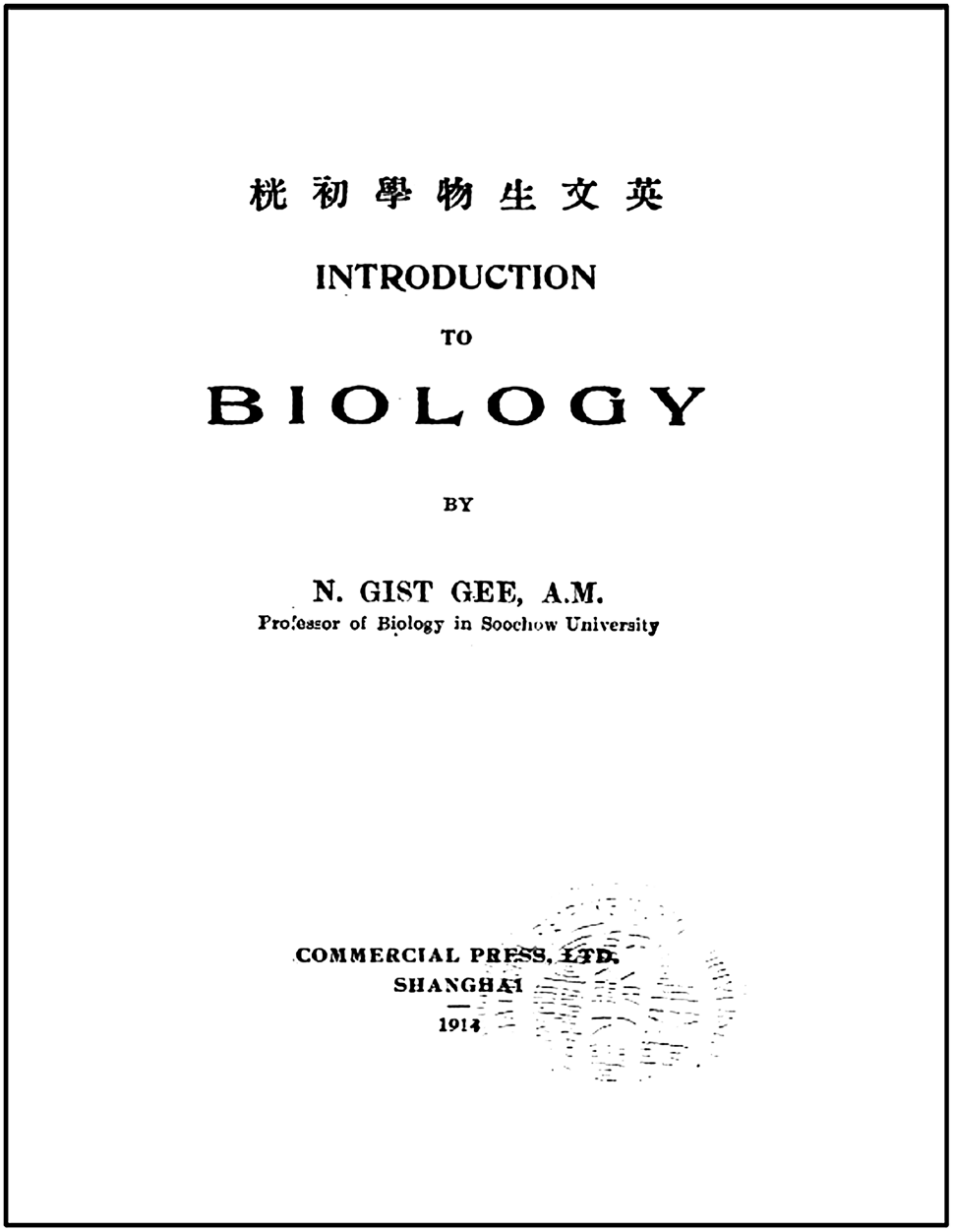
*Introduction to Biology,* by N. Gist Gee

Since the beginning of his college career, Gist Gee was interested in plant and animal taxonomy. During his spare time, he traveled to investigate and collect plant and animal specimens so that he could instruct his students with local biological resources, thus advancing the development of biology taxonomy in China. He published *Catalogue of Plants in Jiangsu Province* in *The National Review* from 1913 and in *Science* from 1919-1921. Gist Gee’s publications in *Science* were translated by Chien Shung-Shu (钱崇澍) and published by the Science Society of China. Additionally, most of the book was appended to *A Textbook of Botany* cited above.

N. Gist Gee was keen to aquatic organisms and hoped to found an institute to research fresh-water organisms. He exchanged a lot of fresh-water sponge specimens with foreign biologists and museums and also published dozens of articles in this research field. Through his work and articles, Chinese biological resources gradually became known by the world, and N. Gist Gee became a well-known authority on fresh-water sponges. His work was succeeded by Wu Chenfu Francis and his other students.

N. Gist Gee was interested in birds as well, and he compiled *A Key to the Birds of Lower Yangtse Valley* and *A Tentative List of Chinese Birds* with Lacy I. Moffett and others. His work set examples for latter research. He also published several articles on Chinese amphibians, reptiles, mammals and other animals, with a few of the articles on amphibians accomplished with A. M. Boring from Yenching University (Zheng, 2015).

N. Gist Gee regarded it very important to the institutionalization of biology research in modern China. He joined the Science Society of China to connect with Chinese scientists, and also launched the Peking Society of Natural History with A. G. Grabau (葛利普), Chi Ping (秉志) and other scientists in 1925. They distributed a magazine and published many research articles. At the same time, they were active for the popularity of science. As secretary and president of the society, Gist Gee worked hard to communicate with scientists at home and abroad. He made the society an important research and communication platform for naturalists.

When he worked for the Rockefeller Foundation, N. Gist Gee made great efforts to promote the establishment of Fan Memorial Institute of Biology. The institute was set up in 1928 by Chi Ping and Hu Hsen-hsu (胡先骕), and N. Gist Gee was the only foreign member of the board.

N. Gist Gee was a great scientist and made outstanding contributions to the development of biology education and research of China. He and his work were important in the transmission of modern biology to China.
